# The impacts of shape factor and heat transfer on two-phase flow of nano and hybrid nanofluid in a saturated porous medium

**DOI:** 10.1038/s41598-022-26169-z

**Published:** 2022-12-18

**Authors:** P. V. Ananth Subray, B. N. Hanumagowda, S. V. K. Varma, Mohammad Hatami

**Affiliations:** 1 School of Applied Sciences, REVA University, Bengaluru, Karnataka India; 2 Mechanical Engineering Department, Ferdowsi University of Mashhad, Mashhad, Iran; 3 Mechanical Engineering Department, Esfarayen University of Technology, Esfarayen, North Khorasan Iran

**Keywords:** Applied mathematics, Computational science

## Abstract

The focus of this article is to obtain the effect of shape factor of the hybrid nanoparticles on the convective heat and mass transference of two immiscible fluids in an inclined duct by employing the perturbation technique. The hybrid nanoparticle of Carbon Nanotube & Sodium alginate is being used with Silicon oil as the base fluid to study the heat and mass phenomena due to the soret effect, viscous dissipation, Darcy and Thermal diffusion. The physical flow problem is then modelled into a set of differential equations. The system of equations is solved analytically to obtain various graphical and numerical results for analyzing the impact of various material parameters on velocity and thermal field. The heat transfer rate and skin friction analysis for the flow dynamics are also investigated. It is observed that the shape factor enhances the fluid flow and temperature distribution. In specific lamina shape particles have better performance comparatively, significance of the soret number can also be observed.

## Introduction

In recent days, the strive to enhance the thermal conductivity of fluids is more and new kinds of fluids namely nanofluids have been developed to fulfil the needs^1–5^. Nanoparticles are colloidal systems with the size of the particles to be $10^{ - 9}$. Industries such as lubricants, hydraulic oils, food industry, solar collectors etc.^6–12^ attract these kinds of fluid which have special properties. Although nanofluids fulfil the needs of these industries in recent days there is a need for better fluids which enhances the heat transfer rates^13–16^. This works deals with the study of the effect of hybrid nanofluids as a lubricant or hydraulic fluid which has better performance than the existing class of fluids.

Hybrid nanofluid is extensively used in various heat transfer applications which include heat exchangers, heat sinks, heat pipes, photovoltaic modules, natural convection enclosures, refrigeration systems, jet impingement cooling, boiling, and thermal energy storage systems. By considering these applications Jana et al.^17^ experimentally obtained the hybrid nanofluids by appending a couple of diverse nanoparticles in the base fluid, as they are a pioneering class of fluid. They noticed the enhancement of heat transfer in these fluids by adding both single and hybrid Nanoparticles. Niihara^18^ examined the composite layer of nanoparticle and concluded that the composite layer not only improves the thermal conductivity but also they enhance the mechanical properties. Chamkha et al.^19^ recorded the heat transfer characteristic of the fluid flow with copper and aluminium oxide filled with water in a porous cavity. The study of hybrid nanofluids in various geometries^20–24^ is studied due to the applications from various researchers.

Many recent studies show the shape factor of the nanoparticle plays a major role in the change in the thermophysical properties with the expected results^25^. Sobamowo^26^ examined the shape factor effect in hybrid nanofluids by varying the values of the Prandtl number. They noticed the desired outcome is obtained in a lamina shape. Hussain et al.^27^ investigated the effect of shape factors in nanoparticles with thermal radiation. Muneeshwaran et al.^28^ examined the role of hybrid nanofluids in the enhancement of heat transfer and their applications in industries.

Another method to enhance the heat transfer rate is by equipping the permeable medium^29^. For example, the use of metal-based porous materials in channel and heat exchangers. The method of using hybrid nanofluid and permeable medium has been investigated by many researchers^30–32^. As permeable media provides more area of contact between the surfaces. Also, hybrid nanofluids improve thermal conduction. Therefore, combining permeable medium and hybrid nanofluids can enhance the productivity of the thermal system. Anwar et al. studied the effect of a porous medium in two-phase flow for electrically conducting and non-conducting fluids^33^.

The fluid flow in two regions is due to the drag force^34^ of the shear stress by fluid flowing next it to, which has a wide range of applications such as oil–gas mixtures, evaporators, boilers, condensers, submerged combustion systems, sewerage treatment plants, air-conditioning and refrigeration plants, and cryogenic plants. The study of two immiscible fluids in different geometries was deliberated by Khaled^35^, Umavathi^36^ and Chen^37^. Two immiscible fluids with one conducting and another non-conducting property were considered by Malashetty et al.^38^. The investigation of two-phase flow in a permeable medium with an electrically conducting and heat-generating or absorbing was carried out by Chamkha^39^. The heat transfer of a two-phase unsteady flow of nanofluids with magnetic properties was investigated by Sheikholeslami et al.^40^ and studied the variations caused due to the haphazard motion of the particle and soret effect using the numerical technique. Some of the recent study related to two-phase flow is carried out^41–45^ in the presence of Casson and micropolar fluids.

From an overview of available literature, very sparse research is carried out related to the hybridization of Carbon Nanotube & Sodium alginate in Silicon oil along with consideration of the shape factor, even though they are widely used as lubricating oil and hydraulic fluid. Hence this study focuses on studying them along with the soret effect and permeable medium in a two-phase flow.

## Problem description

**Figure 1:**
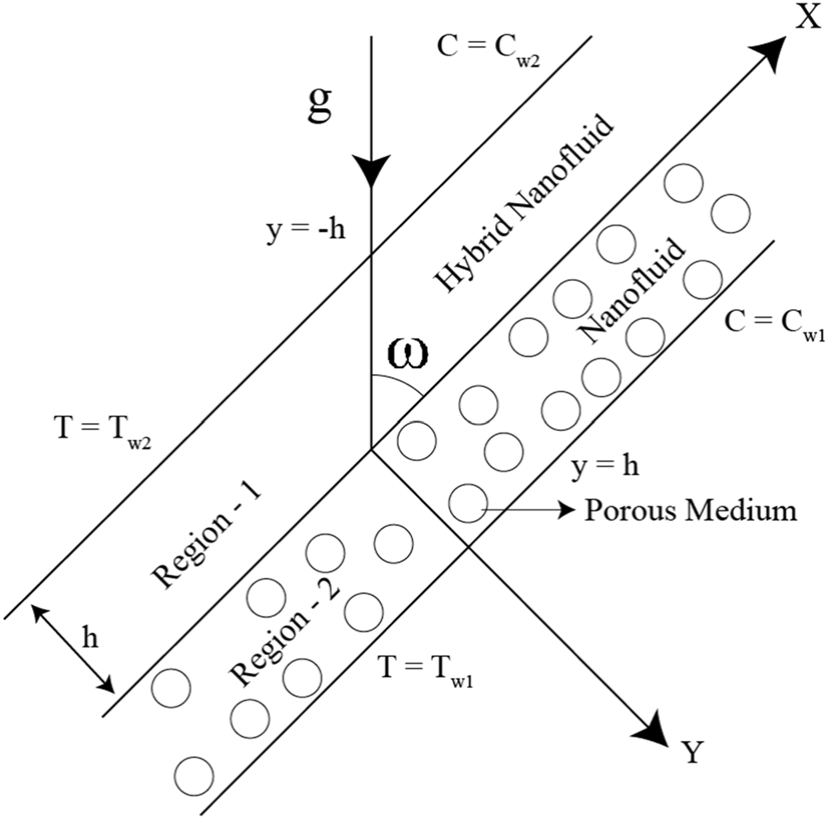
Physical model.

The physical configuration is illustrated in Fig. 1. A steady, laminar, incompressible, fully developed and mixed convective flow of viscous incompressible Hybrid nanofluid along an infinitely long inclined plate with an acute angle $\Theta$ considered, with an x-axis is along the plate with a distance of $h$ between the plates. The left boundary walls are kept at a $T_{w2}$ and the right wall is kept at $T_{w1}$ a temperature where $T_{w1} \ge T_{w2}$. We have assumed the density to be persistent everywhere except when it is multiplied by gravity i.e., Oberbeck-Boussinesq approximation^46^. The flow is assumed to be due to the buoyancy and pressure forces.

We consider Hybrid nanofluid in region-1 $\left( { - h \le y \le 0} \right)$ and Nanofluid in region-2 $\left( {0 \le y \le h} \right)$. We have considered Silicon oil as the base fluid and Carbon Nanotube & Sodium alginate as nanoparticles. Silicon oil is used as a cooling and insulting liquid for transformers and other electrical equipment. The shape factor of the nanoparticle is considered in our study which is presented in Table 1.

**Table 1:** Values of the nanoparticle's Shape factor^47,48^.

Shape of the nanoparticles	Shape factor (q)
Brick	3.7
Blade	8.6
Laminar	16.1576

With these assumptions, Tiwari and Das^49^ model for nanofluids and Darcy law^50^ which excludes the porous medium inertia effects^51,52^ as the flow is unidirectional and function of y. Governing equations of motion, energy and concentration become (Ghasemi and Aminossadati^53^, Muthtamilselvan et al.^54^, Vajravelu et al.^55^):

### Region-1

1 $$\mu_{hnf} \frac{{d^{2} u_{1} }}{{dy^{2} }} + \left( {\rho_{hnf} g\beta_{hnf} } \right)\left( {T_{1} - T_{w2} } \right)\cos (\Theta ) + \left( {\rho_{hnf} g\beta_{hnc} } \right)\left( {C_{1} - C_{w2} } \right)\cos (\Theta ) - \frac{\partial p}{{\partial x}} = 0$$

2 $$k_{hnf} \frac{{d^{2} T_{1} }}{{dy^{{\prime}{2}} }} + \mu_{hnf} \left( {\frac{{du_{1} }}{dy}} \right)^{2} = 0$$

3 $$D_{1} \frac{{d^{2} c_{1} }}{{dy^{2} }} + D_{T} \frac{{d^{2} T_{1} }}{{dy^{2} }} = 0$$

### Region-2

4 $$\mu_{nf} \frac{{d^{2} u^{\prime}_{2} }}{{dy^{{\prime}{2}} }} + \left( {\rho_{nf} g\beta_{nf} } \right)\left( {T_{2} - T_{w2} } \right)\cos (\Theta ) + \left( {\rho_{nf} g\beta_{nc} } \right)\left( {C_{2} - C_{w2} } \right)\cos (\Theta ) - \frac{{\mu_{nf} }}{\kappa }u^{\prime}_{2} - \frac{\partial p}{{\partial x}} = 0$$

5 $$k_{{nf}} \frac{{d^{2} T_{2} }}{{dy^{{\prime 2}} }} + \mu _{{nf}} \left( {\frac{{du_{2}^{\prime } }}{{dy^{\prime}}}} \right)^{2} + \frac{{\mu _{{nf}} }}{\kappa }u_{2}^{{\prime \;\;^{2} }} = 0$$

6 $$D_{2} \frac{{d^{2} c_{2} }}{{dy^{2} }} + D_{T} \frac{{d^{2} T_{2} }}{{dy^{2} }} = 0$$

The above-defined governing equations for velocities (1) and (4) require four conditions to solve. Which are drawn from the assumptions made at the walls and by equating the interface constraints. Similarly, the boundary constraints for temperature and concentration are obtained. Which is of the form:

7 $$u_{1} ( - h) = 0,\;u_{1} (0) = u_{2} (0),\;\mu_{hnf} \frac{{du_{1} (0)}}{dy} = \mu_{nf} \frac{{du_{2} (0)}}{dy},\;u_{2} (h) = 0$$

8 $$T_{1} ( - h) = T_{w2} ,\;T_{1} (0) = T_{2} (0),\;k_{hnf} \frac{{dT_{1} (0)}}{{dy^{\prime}}} = k_{nf} \frac{{dT_{2} (0)}}{{dy^{\prime}}},\;T_{2} (h) = T_{w1}$$

9 $$c_{1} ( - h) = 0,\;c_{1} (0) = c_{2} (0),\;\frac{{dc_{1} (0)}}{dy} = \frac{{D_{2} }}{{D_{1} }}\frac{{dc_{2} (0)}}{dy},\;c_{2} (h) = 1$$

The effective characteristics of hybrid nanofluids and Mono nanofluids considered in our study are listed in Table 2. Where $\phi_{1}$ and $\phi_{2}$ are the solid volume fraction of the particles. $K_{nf}$ and $K_{hnf}$ are the thermal conductivity with the shape factor $q$ according to Maxwell^56^, Brinkman model for the viscosity is considered^57^. The subscripts $hnf$, $nf$, $s$, $1$ and $2$ represents the hybrid nanofluid, nanofluid, solid nanoparticle, first nanoparticle and second nanoparticle respectively.

**Table 2:** Thermophysical properties of nanoparticle and hybrid nanoparticle.

Properties	Mono nanofluid	Hybrid nanofluid
Density	$\rho_{nf} = \left( {1 - \phi } \right)\rho_{f} + \phi \rho_{s}$	$\rho_{hnf} = [(1 - \phi_{1} )\rho_{f} + \phi_{1} \rho_{s1} ](1 - \phi_{2} ) + \rho_{s2} \phi_{2}$
Heat capacity	$\left( {\rho C_{p} } \right)_{nf} = \left( {1 - \phi } \right)\left( {\rho C_{p} } \right)_{f} + \phi \left( {\rho C_{p} } \right)_{s}$	$\left( {\rho C_{p} } \right)_{hnf} = \left( {\rho C_{p} } \right)_{f} \left( {1 - \phi_{2} } \right)\left[ {\left( {1 - \phi_{1} } \right) + \phi_{1} \frac{{\left( {\rho C_{p} } \right)_{s1} }}{{\left( {\rho C_{p} } \right)_{f} }}} \right] + \phi_{2} \left( {\rho C_{p} } \right)_{s2}$
Dynamic viscosity^57^	$\mu_{nf} = \frac{{\mu_{f} }}{{\left( {1 - \phi } \right)^{2.5} }}$	$\mu_{hnf} = \frac{{\mu_{f} }}{{\sqrt {\left( {1 - \phi_{1} } \right)^{5} \left( {1 - \phi_{2} } \right)^{5} } }}$
Thermal conductivity^56^	$K_{nf} = K_{f} \left\{ {\frac{{K_{s1} + \left( {q - 1} \right)K_{f} - \left( {q - 1} \right)\phi \left( {K_{f} - K_{s1} } \right)}}{{K_{s1} + \left( {q - 1} \right)K_{f} + \phi \left( {K_{f} - K_{s1} } \right)}}} \right\}$	$k_{hnf} = k_{hf} \left[ {\frac{{\left( {k_{s2} + \left( {q - 1} \right)k_{nf} } \right) - \left( {q - 1} \right)\phi_{n2} \left( {k_{nf} - k_{s2} } \right)}}{{\left( {k_{s2} + \left( {q - 1} \right)k_{nf} } \right) + \phi_{n2} \left( {k_{nf} - k_{s2} } \right)}}} \right]$

To convert the Eqs. (1)–(9) to dimensionless form we use the following terms:10

Substituting Eq. (10) into Eqs. (1)–(9) and after dropping the asterisks we obtain the following non-dimensional form of the governing equations considering Supplementary Information for constant parameters.

### Region-1

11 $$\frac{{d^{2} u_{1} }}{{dy^{2} }} + A_{1} Grl\theta_{1} + A_{2} Grc\psi_{1} - P_{2} = 0$$

12 $$\frac{{d^{2} \theta_{1} }}{{dy^{2} }} + BrA_{4} \left( {\frac{{du_{1} }}{dy}} \right)^{2} = 0$$

13 $$\frac{1}{{S_{c} }}\frac{{d^{2} \psi_{1} }}{{dy^{2} }} + S_{T} \frac{{d^{2} \theta_{1} }}{{dy^{2} }} = 0$$

### Region II

14 $$\frac{{d^{2} u_{2} }}{{d^{2} y}} + \left( {A_{5} Grl\theta_{2} } \right) + \left( {A_{6} Grc\psi_{2} } \right) - \sigma^{2} u_{2} + P_{1} = 0$$

15 $$\frac{{d^{2} \theta_{2} }}{{dy^{2} }} + A_{7} B_{r} \left\{ {\left( {\frac{{du_{2} }}{dy}} \right)^{2} + \sigma^{2} u_{2}^{2} } \right\} = 0$$

16 $$\frac{1}{{S_{c} }}\frac{{d^{2} \psi_{2} }}{{dy^{2} }} + S_{T} \frac{{d^{2} \theta_{2} }}{{dy^{2} }} = 0$$

The non-dimensional boundary and interface conditions are17

### Solution method

The Governing equations of assumed flow replica & respective boundary conditions are defined in Eqs. (11)–(17), which are ordinary nonlinear coupled equations. Where the Brinkman number is assumed to be perturbation constant to overcome limitations of the assumed analytical technique. Brinkman number has lots of practical applications when the value ranges between zero and one. By considering the special properties of a Brinkman number, we have calculated the approximations of temperature, concentration and velocity:

18 $$u_{i} (y) = u_{i0} (y) + Bru_{i1} + \left( {Br} \right)^{2} u_{i2} (y) + \ldots$$

19 $$\theta_{i} (y) = \theta_{i0} (y) + Br\theta_{i1} + \left( {Br} \right)^{2} \theta_{i2} (y) + \ldots$$

20 $$\psi_{i} (y) = \psi_{i0} (y) + Br\psi_{i1} + \left( {Br} \right)^{2} \psi_{i2} (y) + \ldots$$

Inputting the Eqs. (18)–(20) into (12)–(18) and equating the coefficients of the like powers of Br to zero, we obtain the following equations:

### Zeroth order equations

#### Region-1

21 $$\frac{{d^{2} u_{10} }}{{dy^{2} }} + A_{1} Grl\theta_{10} + A_{2} Grc\psi_{10} - P_{2} = 0$$

22 $$\frac{{d^{2} \theta_{10} }}{{dy^{2} }} = 0$$

23 $$\frac{1}{{S_{c} }}\frac{{d^{2} \psi_{10} }}{{dy^{2} }} + S_{T} \frac{{d^{2} \theta_{10} }}{{dy^{2} }} = 0$$

#### Region-2

24 $$\frac{{d^{2} u_{20} }}{{d^{2} y}} + \left( {A_{5} Grl\theta_{20} } \right) + \left( {A_{6} Grc\psi_{20} } \right) - \sigma^{2} u_{20} + P_{1} = 0$$

26 $$\frac{{d^{2} \theta_{20} }}{{dy^{2} }} = 0$$

26 $$\frac{1}{{S_{c} }}\frac{{d^{2} \psi_{20} }}{{dy^{2} }} + S_{T} \frac{{d^{2} \theta_{20} }}{{dy^{2} }} = 0$$

The boundary and interface conditions for zeroth order are

27 $$\begin{gathered} u_{10} ( - 1) = 0,u_{10} (0) = u_{20} (0),\frac{{du_{10} (0)}}{dy} = \frac{{\mu_{nf} }}{{\mu_{hnf} }}\frac{{du_{20} (0)}}{dy},u_{20} (1) = 0 \hfill \\ \theta_{10} ( - 1) = 0,\theta_{10} (0) = \theta_{20} (0),\frac{{d\theta_{10} (0)}}{dy} = \frac{{k_{nf} }}{{k_{hnf} }}\frac{{d\theta_{20} (0)}}{dy},\theta_{20} (1) = 1 \hfill \\ \psi_{10} ( - 1) = 0,\psi_{10} (0) = \psi_{20} (0),\frac{{d\psi_{10} (0)}}{dy} = \frac{{D_{2} }}{{D_{1} }}\frac{{d\psi_{20} (0)}}{dy},\psi_{20} (1) = 1 \hfill \\ \end{gathered}$$

### First-order equations

#### Region-1

28 $$\frac{{d^{2} u_{11} }}{{dy^{2} }} + A_{1} Grl\theta_{11} + A_{2} Grc\psi_{11} = 0$$

29 $$\frac{{d^{2} \theta_{11} }}{{dy^{2} }} + A_{4} \left( {\frac{{du_{10} }}{dy}} \right)^{2} = 0$$

30 $$\frac{1}{{S_{c} }}\frac{{d^{2} \psi_{11} }}{{dy^{2} }} + S_{T} \frac{{d^{2} \theta_{11} }}{{dy^{2} }} = 0$$

#### Region-2

31 $$\frac{{d^{2} u_{21} }}{{d^{2} y}} + \left( {A_{5} Grl\theta_{21} } \right) + \left( {A_{6} Grc\psi_{21} } \right) - \sigma^{2} u_{21} + P_{1} = 0$$

32 $$\frac{{d^{2} \theta_{21} }}{{dy^{2} }} + A_{7} \left\{ {\left( {\frac{{du_{20} }}{dy}} \right)^{2} + \sigma^{2} u_{20}^{2} } \right\} = 0$$

33 $$\frac{1}{{S_{c} }}\frac{{d^{2} \psi_{21} }}{{dy^{2} }} + S_{T} \frac{{d^{2} \theta_{21} }}{{dy^{2} }} = 0$$

The boundary and interface conditions for the first order are34

Equations (21)–(34) are solved by integrating and using the respective boundary conditions we get the following solution. The intermediate steps are vomited as it’s a simple integration and simplification.

#### Temperature

35 $$\theta_{1} = c_{1} y + c_{2} + Br\left( {L_{5} \frac{{y^{6} }}{120} + L_{6} \frac{{y^{5} }}{20} + L_{7} \frac{{y^{4} }}{12} + L_{8} \frac{{y^{3} }}{6} + L_{911} \frac{{y^{2} }}{2} + c_{11} y + c_{12} } \right)$$

36 $$\theta_{2} = c_{3} y + c_{4} - Br\left( {A_{7} \left[ \begin{gathered} \frac{{L_{9} }}{{4\sigma^{2} }}{\text{Cosh}} 2\sigma y + \frac{{L_{10} }}{{4\sigma^{2} }}{\text{Sinh}} 2\sigma y + \frac{{L_{11} }}{{\sigma^{3} }}y\sigma {\text{Sinh}} \sigma y + \frac{{L_{12} }}{{\sigma^{3} }}y\sigma {\text{Cosh}} \sigma y + L_{17} \frac{{y^{2} }}{2} \hfill \\ + \left( {\frac{{L_{14} }}{\sigma } - \frac{{L_{12} }}{{\sigma^{2} }} - \frac{{L_{12} }}{{\sigma^{2} }}} \right)\frac{1}{\sigma }{\text{Sinh}} \sigma y + \left( {\frac{{L_{13} }}{\sigma } - \frac{{L_{11} }}{{\sigma^{2} }} - \frac{{L_{11} }}{{\sigma^{2} }}} \right)\frac{1}{\sigma }{\text{Cosh}} \sigma y + L_{15} \frac{{y^{4} }}{12} + L_{16} \frac{{y^{3} }}{6} \hfill \\ \end{gathered} \right] + c_{21} y + c_{22} } \right)$$

#### Velocity

$$u_{1} = \left( {L_{1} \frac{{y^{3} }}{6} + L_{2} \frac{{y^{2} }}{2} + b_{11} y + b_{12} } \right) + Br\left( {\frac{{L_{32} }}{56}y^{8} + \frac{{L_{33} }}{42}y^{7} + \frac{{L_{34} }}{30}y^{6} + \frac{{L_{35} }}{20}y^{5} + \frac{{L_{36} }}{12}y^{4} + \frac{{L_{37} }}{6}y^{3} + \frac{{L_{38} }}{2}y^{2} + c_{51} y + c_{52} } \right)$$

37 $$u_{2} = \left( \begin{gathered} b_{21} {\text{Cosh}} \sigma y + b_{22} {\text{Sinh}} \sigma y \hfill \\ + L_{3} y + L_{4} \hfill \\ \end{gathered} \right) + Br\left( \begin{gathered} c_{61} {\text{Cosh}} \sigma y + c_{62} {\text{Sinh}} \sigma y + L_{39} {\text{Cosh}} 2\sigma y + L_{40} {\text{Sinh}} 2\sigma y \hfill \\ + L_{41} \left( {\frac{{y^{2} }}{2}{\text{Cosh}} \sigma y - \frac{y}{2\sigma }{\text{Sinh}} \sigma y} \right) + L_{42} \left( {\frac{{y^{2} }}{2}{\text{Sinh}} \sigma y - \frac{y}{2\sigma }{\text{Cosh}} \sigma y} \right) \hfill \\ + L_{43} \frac{y}{2\sigma }{\text{Cosh}} \sigma y + L_{44} \frac{y}{2\sigma }{\text{Sinh}} \sigma y + L_{45} \left( {y^{4} + \frac{12}{{\sigma^{2} }}y^{2} + \frac{24}{{\sigma^{4} }}} \right) \hfill \\ + L_{46} \left( {y^{3} + \frac{6}{{\sigma^{2} }}y} \right) + L_{47} \left( {y^{2} + \frac{2}{{\sigma^{2} }}} \right) + L_{48} y + L_{49} \hfill \\ \end{gathered} \right)$$

#### Concentration

$$\psi_{1} = c_{5} y + c_{6} + Br\left( {L_{18} y^{6} + L_{19} y^{5} + L_{20} y^{4} + L_{21} y^{3} + L_{22} y^{2} + c_{31} y + c_{32} } \right)$$

38 $$\psi_{2} = c_{7} y + c_{8} + Br\left( \begin{gathered} L_{23} {\text{Cosh}} 2\sigma y + L_{24} {\text{Sinh}} 2\sigma y + L_{25} y\sigma {\text{Sinh}} \sigma y + L_{26} y\sigma {\text{Cosh}} \sigma y + L_{27} {\text{Sinh}} \sigma y \hfill \\ + L_{28} {\text{Cosh}} \sigma y + L_{29} y^{4} + L_{30} y^{3} + L_{31} y^{2} + c_{41} y + c_{42} \hfill \\ \end{gathered} \right)$$

#### Derived quantities

Nusselt number: It is the rate of heat transfer at the right & left plate.

39 $$\left( {Nu} \right)_{y = - 1} = \left( {\frac{{d\theta_{1} }}{dy}} \right)_{y = - 1} = c_{1} + Br\left( {c_{11} - \frac{{L_{5} }}{20} + \frac{{L_{6} }}{4} - \frac{{L_{7} }}{3} + \frac{{L_{8} }}{2} - L_{911} } \right)$$

40 $$\left( {Nu} \right)_{y = 1} = - \left( {\frac{{d\theta_{2} }}{dy}} \right)_{y = 1} = - c_{1} - Br\left( {c_{11} + \frac{{L_{5} }}{20} + \frac{{L_{6} }}{4} + \frac{{L_{7} }}{3} + \frac{{L_{8} }}{2} + L_{911} } \right)$$

Skin friction: It is friction between the fluid and the surface of a solid for fluid in relative motion.

41 $$(\tau )_{y = - 1} = \left( {\frac{{du_{1} }}{dy}} \right)_{y = - 1} = \left( {b_{11} + \frac{{L_{1} }}{2} - L_{2} } \right) + Br\left( {c_{51} - \frac{{L_{32} }}{7} + \frac{{L_{33} }}{6} - \frac{{L_{34} }}{5} + \frac{{L_{35} }}{4} - \frac{{L_{36} }}{3} + \frac{{L_{37} }}{2} - L_{38} } \right)$$

42 $$(\tau )_{y = 1} = - \left( {\frac{{du_{2} }}{dy}} \right)_{y = 1} = - \left( {b_{11} + \frac{{L_{1} }}{2} + L_{2} } \right) - Br\left( {c_{51} + \frac{{L_{32} }}{7} + \frac{{L_{33} }}{6} + \frac{{L_{34} }}{5} + \frac{{L_{35} }}{4} + \frac{{L_{36} }}{3} + \frac{{L_{37} }}{2} + L_{38} } \right)$$

## Results and discussion

An analytical method is used to calculate the two-layered hybrid nanofluid flow by employing regular perturbation techniques for numerous germane parameters. The flow is formulated by considering Silicon oil as a base fluid and CNT and $\left( {C_{6} H_{8} O_{6} } \right)_{n}$ nanoparticles. Graphical representation of the solution is given by graphs and Eqs. (2)–(19) which furnish special features of saturated hybrid nanofluid in two-immiscible fluids in presence of soret effect and angle of inclination.

**Figure 2:**
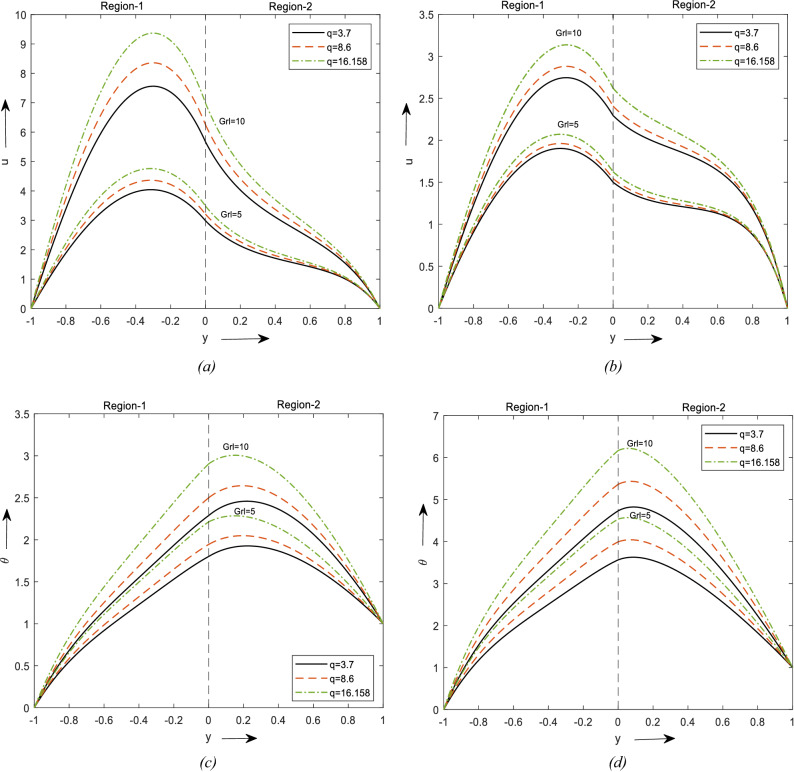
Velocity profiles for different values of Thermal Grashof number. (**a**) For two different values of $\phi$. (**b**) for a value of $\phi$. Temperature profiles for different values of Br. (**c**) For two different values of $\phi$. (**d**) For a value of $\phi$.

Figure 2a represents the change in velocity with Grl and shape factor. For increasing values of Gr and an increment in the velocity of fluid flow can be observed, maximum can be observed in region-1 and then reduces at region-2. Region-1 consists of a hybrid nanofluid and region-2 with nanofluid in the occurrence of a saturated permeable medium and hence that difference in the velocity is noticed. It can be noticed that the velocity for the Laminar shape is greater than the blade shape and brick-shaped nanoparticles.

The same results are observed for temperature in Fig. 2c. Figure 2b and d depicts the change in velocity & temperature due to the influence of Gr and solid volume fraction. We have considered the $\phi_{2}$ to be zero and obtained Fig. 2b with this the region-1 reduces to the Nanofluid and region-2 reduces to clear fluid in the presence of a saturated porous medium. It can be observed that the Laminar shaped nanoparticle has a higher velocity in region-1 and lesser in region-2 to due the porosity of the material.

**Figure 3:**
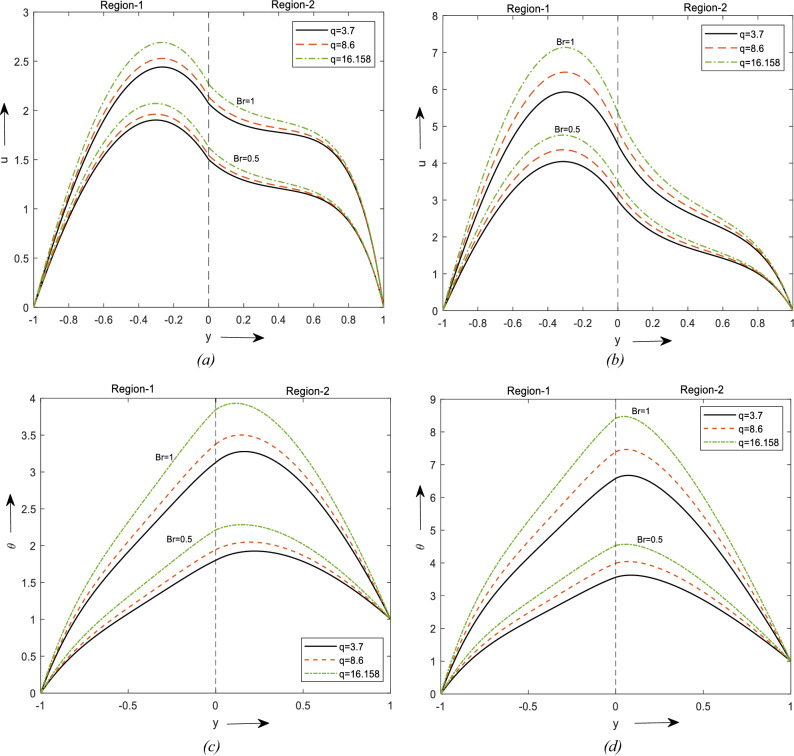
Velocity profiles for different values of Br. (**a**) For two different values of $\phi$. (**b**) For a value of $\phi$. Temperature profiles for different values of Br. (**c**) For two different values of $\phi$. (**d**) For a value of $\phi$.

Figure 3a and b shows the effect of Br on the velocity of the fluid flow. In Fig. 3a it is spotted that velocity increment is more prevalent in region-1 than region-2 and Fig. 3b shows that the velocity is higher in the presence of nanofluid than in the clear fluid. It is obtained by keeping $\phi_{2}$ to zero. From Figure 3c and d it can be noticed that the temperature increases by incrementing the values of Br. The increment of fluid flow and temperature can be witnessed due to the enhancement of the thermal energy produced due to viscous dissipation. The dissipation of upwards fluid temperature and subsequently the buoyancy force. Therefore, an increment in the buoyancy force upsurges the fluid flow in the upward direction. From these graphs, it can be noticed the velocity and temperature are higher for laminar shape nanoparticles followed by blade and brick shape.

**Figure 4:**
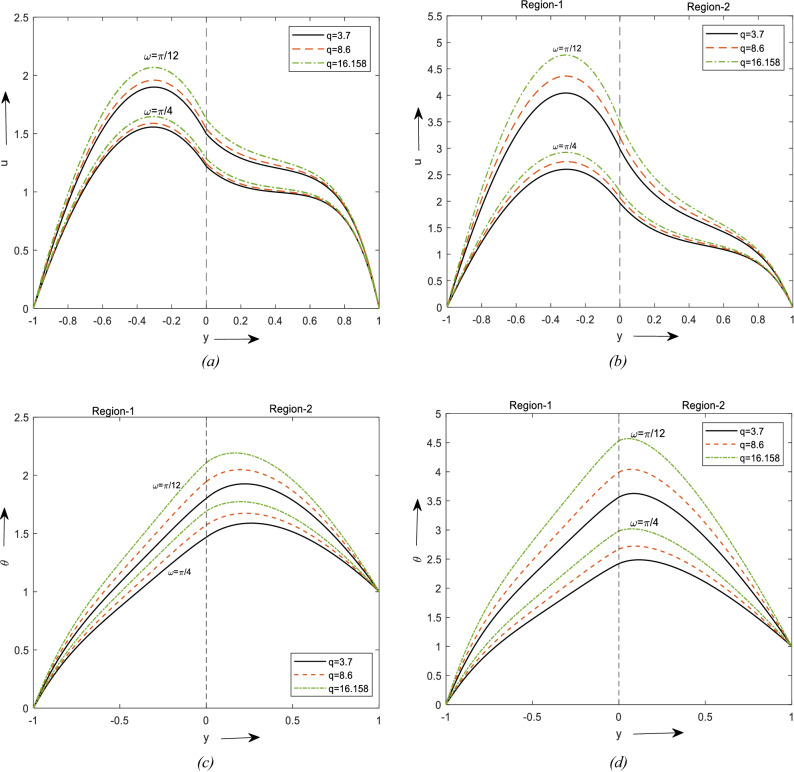
Velocity profiles for different values of $\omega$. (**a**) For two different values of $\phi$. (**b**) For a value of $\phi$. Temperature profiles for different values of $\omega$. (**c**) For two different values of $\phi$. (**d**) For a value of $\phi$.

Figure 4a–d demonstrates the effect of the angle of inclination on fluid velocity and temperature for both regular $\left( {\phi = 0} \right)$ and nanofluid $\left( {\phi \ne 0} \right)$. It can be seen that the velocity of the fluid is decreased by incrementing the angle and it can also be observed that for velocity is higher for the hybrid nanofluid than for the mono nanofluid. Whereas the temperature is wise versa it is due to the increase of amplification and driving force acting on the fluid. Also, the shape of the nanoparticle chosen plays an important role and is observed maximum for lamina-shaped particles.

**Figure 5:**
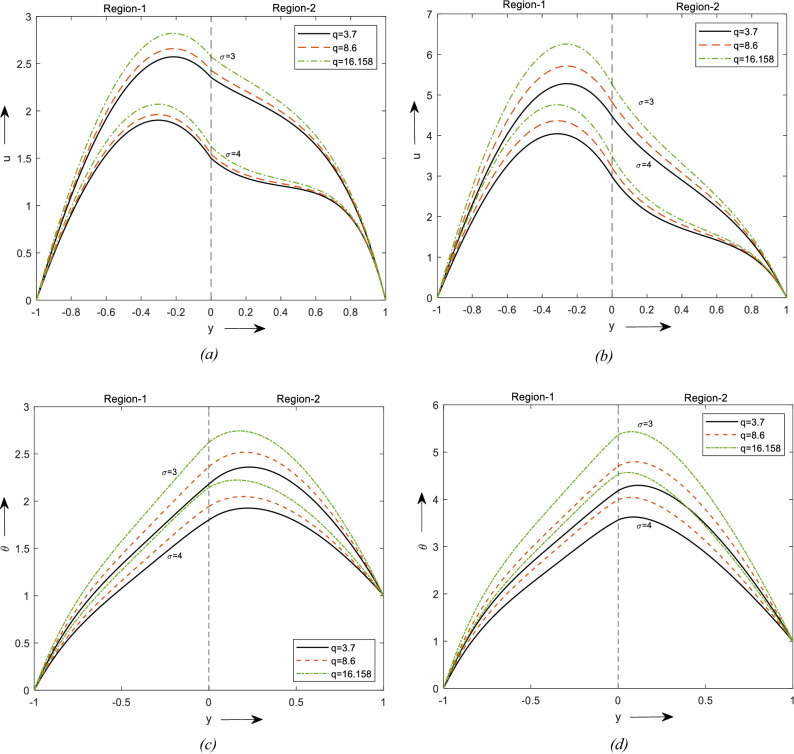
Velocity profiles for different values of $\sigma$. (**a**) For two different values of $\phi$. (**b**) For a value of $\phi$. Temperature profiles for different values of $\sigma$. (**c**) For two different values of $\phi$. (**d**) For a value of $\phi$.

The influence of the porosity medium parameter $\left( \sigma \right)$ on velocity and temperature distribution can be seen in Fig. 5a–d for CNT and cobalt ferrite in silicon oil. It is perceived that the increase $\sigma$ reduces the fluid flow and temperature. Whereas velocity is observed to be greater in region-1 than in region-2. Also, when $\phi_{2}$ is kept at zero it can be observed velocity is higher for smaller values $\sigma$ and then reduces in the clear fluid region. The Laminar shaped particles have more velocity and temperature than others. It is quite seeming to be unusual, but the friction is caused by flow and porous media so thermal conductivity raises which results in enhancement of temperature can be observed in the above figure and the other trends are the same as mentioned for velocity.

**Figure 6:**
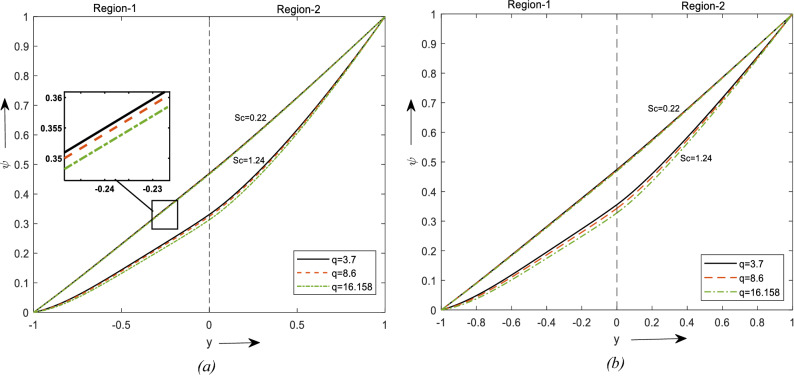
Concentration profiles for different values of Sc. (**a**) For two different values of $\phi$. (**b**) For a value of $\phi$.

Figure 6a and b are the plots of concentration for various values of Sc and shape factor. Comparing the arches from the figure, it can be observed that an increment in Schmidt number declines the concentration at all the points. This is due to the concentration buoyancy effect which reduces fluid flow. Further concentration can be reduced by employing laminar-shaped nanoparticles. Similar phenomena can be observed in Fig. 6b in the absence of a hybrid nanofluid.

**Figure 7:**
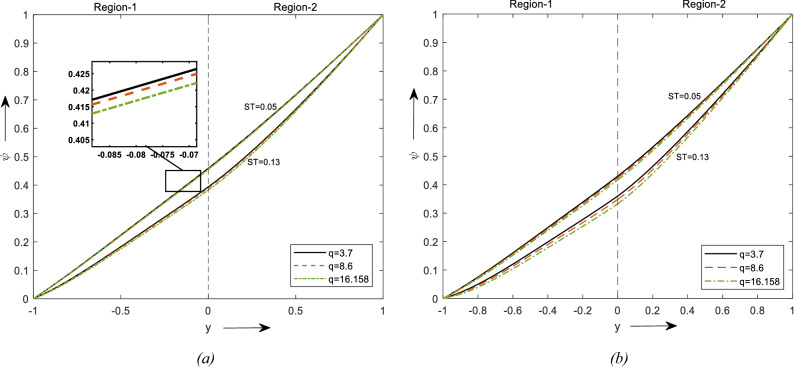
Concentration profiles for different values of ST. (**a**) For two different values of $\phi$. (**b**) For a value of $\phi$.

Figure 7a and b shows the graph for increasing values of thermophoresis to concentration. It depicts the impact of temperature gradient on mass diffusion. In graphs, an increase in Thermophoresis provokes a rise in temperature and fluid concentration decays. Thermophoresis is observed to be utmost in when nanoparticles region and minimal in the hybrid nanofluid region. Concentration is observed to be maximum for the Brick shaped particles which are followed by Blade and laminar. A similar trend is observed when region-1 is considered a nanofluid region and other to be a clear fluid region.

**Figure 8:**
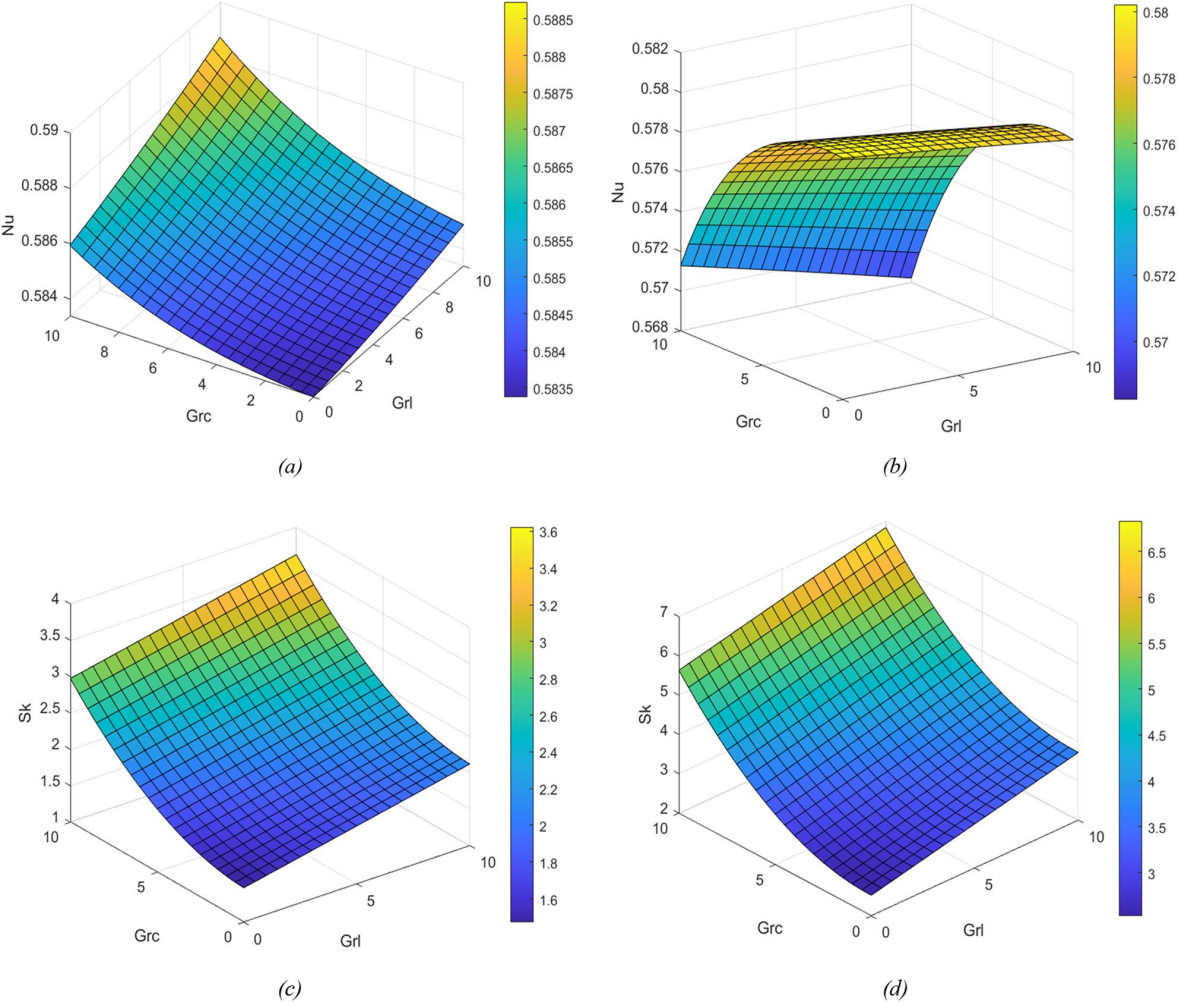
Nusselt graphs for change in GrL and GrC (**a**) at the left plate (y = − 1) (**b**) at the right plate (y = 1). Skin friction for change in GrL and GrC (**c**) at the left plate (y = − 1) (**d**) at the right plate (y = 1).

Figure 8 depicts the Nu and Sk for different values of Grc and GrL at the plates. Figure 8a is plotted for the nusselt number at y = − 1 and Figure 8b at y = 1, Figure 8c is plotted for skin friction number at y = − 1 and Figure 8d at y = 1. From these graphs, an increasing value of Grc and GrL, a rise in the nusselt number is observed. In Figure 8a when the GrL is kept to be constant and for increasing values of Grc, the rate of heat transfer reaches the peak gradually at y = − 1. From Figure 8b we can notice that heat transfer decrease gradually at the right plate (y = 1). The results can be analysed by keeping the Grc Constant and varying GrL.

**Figure 9:**
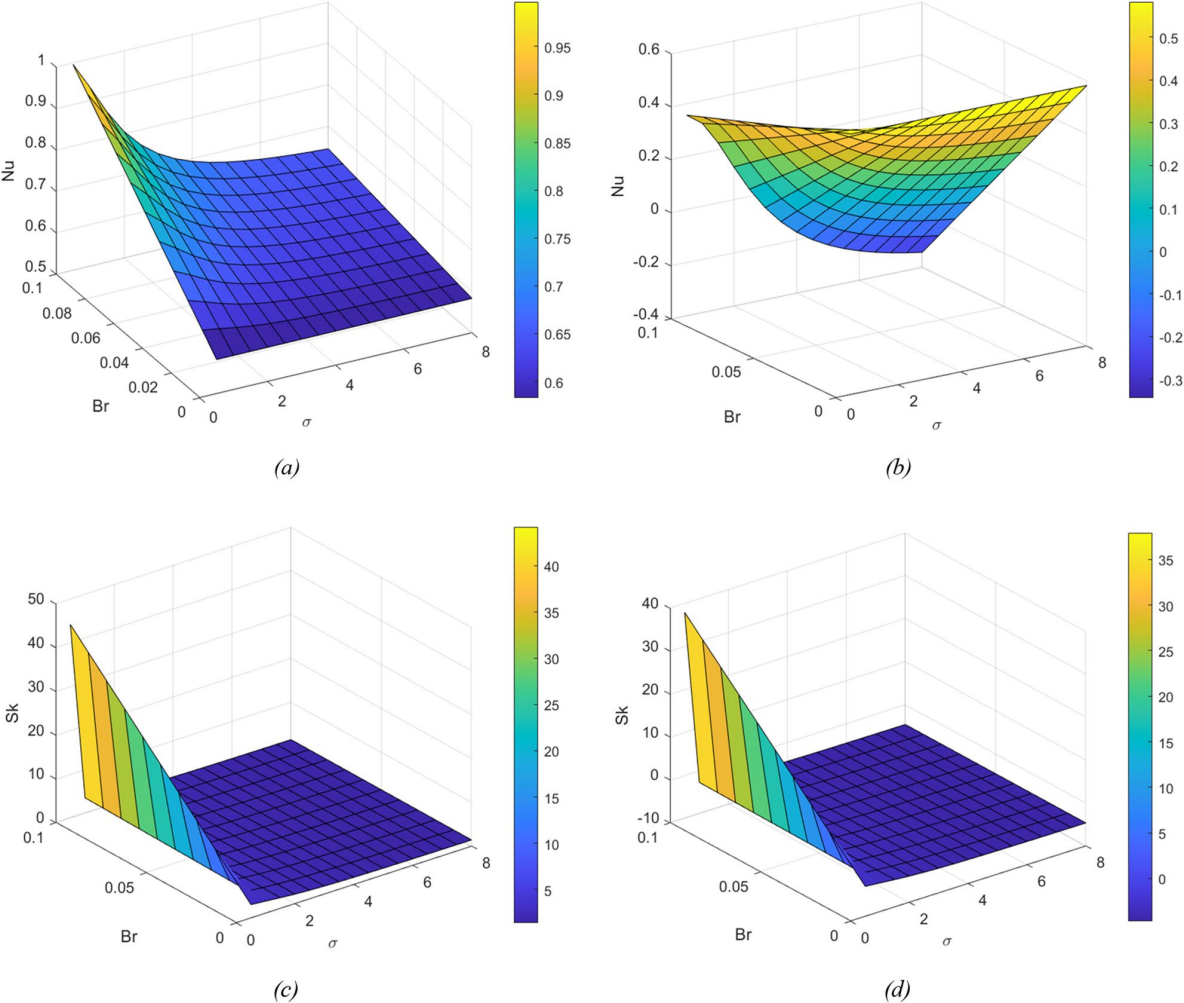
Nusselt graphs for change in Br and $\sigma$ (**a**) at the left plate (y = − 1) (**b**) at the right plate (y =). Skin friction for change in Br and $\sigma$ (**c**) at the left plate (y = − 1) (**d**) at the right plate (y = 1).

To find the combined effect of Br and porous media parameters on heat transfer rate and resistance to the flow, the Nusselt number and Skin friction graphs are plotted, which are displayed in Fig. 9a–d respectively. It is observed that the heat transfer rate and resistance to the flow increase with the augment of Br by keeping $\sigma$ as constant. As Nu increases, heat transfers from the fluid to the wall. A similar observation can be made by keeping Br constant and varying $\sigma$.

### Validation of the results

**Figure 10:**
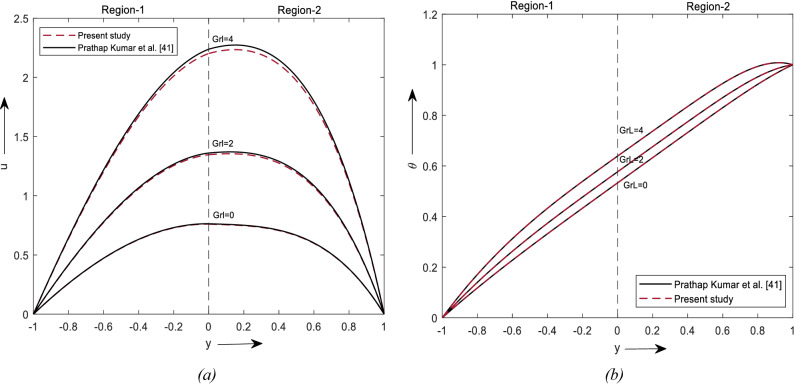
Comparison results of (**a**) velocity profile. (**b**) Temperature profile.

Prathap Kumar et al^58^ considered a two-layer mixed convective flow of viscous fluid in a vertical channel with a chemical reaction. The governing equations Eqs. (1)–(7) of our study show good agreement with the literature of Prathap Kumar et al. in Eq. (2.1)–(2.7), in the absence of Nanofluids and Soret effect in our paper and chemical reaction in their paper. Table 3 shows the comparative study for different values of Brinkman number for velocity and temperature and Fig. 10 shows the comparison of the present work and Prathap Kumar et al. The results of our study are in a similar pattern to them, but not exactly equal to their result, as they have considered chemical reaction parameters to be unity (Supplementary file).

**Table 3:** Fluid flow and temperature values for various values of Br.

Temperature					Velocity				
	Br = 0		Br = 0.5			Br = 0		Br = 0.5	
y	Present	Prathap Kumar et al.^58^	Present	Prathap Kumar et al.^58^	y	Present	Prathap Kumar et al.^58^	Present	Prathap Kumar et al.^58^
− 1	0	0	0	0	− 1	0.0000	0.0000	0.0000	0.0000
− 0.8	0.1000	0.1000	0.1665	0.1790	− 0.8	0.2906	0.2905	0.3200	0.3128
− 0.6	0.2000	0.2000	0.3020	0.3219	− 0.6	0.5314	0.5347	0.5898	0.5763
− 0.4	0.3000	0.3000	0.4171	0.4407	− 0.4	0.7172	0.7265	0.8001	0.7824
− 0.2	0.4000	0.4000	0.5212	0.5459	− 0.2	0.8426	0.8591	0.9421	0.9239
0	0.5000	0.5000	0.6210	0.6458	0	0.9022	0.9260	0.9178	0.9938
0.2	0.6000	0.6000	0.7201	0.7447	0.2	0.8908	0.9198	0.9872	0.9849
0.4	0.7000	0.7000	0.8178	0.8421	0.4	0.8030	0.8329	0.8257	0.8894
0.6	0.8000	0.8000	0.9079	0.9303	0.6	0.6334	0.6570	0.6580	0.6992
0.8	0.9000	0.9000	0.9775	0.9924	0.8	0.3768	0.3828	0.4026	0.4057
1	1	1	1	1	1	0.0000	0.0000	0.0000	0.0000

## Conclusion

- The fluids adjacent to the outer walls of the channel pass slowly, while the fluids with higher temperatures around the interface move with greater velocity.
- Current study shows that using a hybrid nanofluid is an important aspect of the cooling and heating process.
- From the graphs, it is noticed that the Grl and Br enhance the flow and temperature of the fluids, whereas the angle of inclination, and porosity of the medium downgrade.
- The effect of soret number can be significantly observed on velocity and concentration equations.
- A decrement in heat transfer rate at the hot plate and an increment at the cold plot are observed due to viscous dissipation.
- The effect of thermal diffusion is to increase the flow and heat transfer rate in the nanofluid region.
- The concentration of nanoparticles directly affects velocity and temperature in a manner of decreasing and increasing behaviour, respectively, due to their boundary layers.
- The comparison of velocity and temperature with Prathap Kumar et al.^58^ shows that they are in good agreement (Table 3 and Fig. 10).

## Supplementary Information

Supplementary Information.

## Data Availability

The datasets used and/or analysed during the current study are available from the corresponding author upon reasonable request.

## List of symbols

$\omega$: Angle of inclination
$$Br$$: Brinkman number
$$Sc$$: Schmid number
$$ST$$: Soret parameter
$Grl$: Thermal grashof number
$Grc$: Solutal grashof number
$\phi$: Solid volume fraction
$\sigma$: Porous medium parameter
$$g$$: Gravitational force
$$Nu$$: Nusselt number
$$\tau$$: Skin friction
$\rho$: Density of fluid
$$\theta$$: Dimensionless temperature
$$\psi$$: Dimensionless concentration
$\kappa$: Thermal conductivity of a fluid
$$\mu$$: Dynamics viscosity (m^2^s^-1^)
$$v$$: Kinematic viscosity (m^2^s^-1^)
$\beta$: Thermal expansion coefficient
$\phi$: Porosity of the porous media

## Subscripts

$$nf$$: Nanofluid
$$hnf$$: Hybrid nanofluid
$$f$$: Base fluid
$$s$$: Solid particle
