# Supplementary material for: The impacts of shape factor and heat transfer on two-phase flow of nano and hybrid nanofluid in a saturated porous medium
Source: Sci Rep. 2022 Dec 18;12:21864. doi: 10.1038/s41598-022-26169-z (PMC9760659; doi:10.1038/s41598-022-26169-z)
Supplement: Supplementary file 1 — Supplementary Information. [file 41598_2022_26169_MOESM1_ESM.docx]

| **Appendix:** |    |
| --- | --- |
|  |  |
